# Transcriptome Analysis of Scorpion Species Belonging to the *Vaejovis* Genus

**DOI:** 10.1371/journal.pone.0117188

**Published:** 2015-02-06

**Authors:** Verónica Quintero-Hernández, Santos Ramírez-Carreto, María Teresa Romero-Gutiérrez, Laura L. Valdez-Velázquez, Baltazar Becerril, Lourival D. Possani, Ernesto Ortiz

**Affiliations:** 1 Departamento de Medicina Molecular y Bioprocesos, Instituto de Biotecnología, Universidad Nacional Autonóma de México, Cuernavaca, Morelos, México; 2 Facultad de Ciencias Químicas and Facultad de Medicina, Universidad de Colima, Colima, México; CNRS, FRANCE

## Abstract

Scorpions belonging to the Buthidae family have traditionally drawn much of the biochemist’s attention due to the strong toxicity of their venoms. Scorpions not toxic to mammals, however, also have complex venoms. They have been shown to be an important source of bioactive peptides, some of them identified as potential drug candidates for the treatment of several emerging diseases and conditions. It is therefore important to characterize the large diversity of components found in the non-Buthidae venoms. As a contribution to this goal, this manuscript reports the construction and characterization of cDNA libraries from four scorpion species belonging to the *Vaejovis* genus of the Vaejovidae family: *Vaejovis mexicanus*, *V. intrepidus*, *V. subcristatus* and *V. punctatus*. Some sequences coding for channel-acting toxins were found, as expected, but the main transcribed genes in the glands actively producing venom were those coding for non disulfide-bridged peptides. The ESTs coding for putative channel-acting toxins, corresponded to sodium channel β toxins, to members of the potassium channel-acting α or κ families, and to calcium channel-acting toxins of the calcin family. Transcripts for scorpine-like peptides of two different lengths were found, with some of the species coding for the two kinds. One sequence coding for La1-like peptides, of yet unknown function, was found for each species. Finally, the most abundant transcripts corresponded to peptides belonging to the long chain multifunctional NDBP-2 family and to the short antimicrobials of the NDBP-4 family. This apparent venom composition is in correspondence with the data obtained to date for other non-Buthidae species. Our study constitutes the first approach to the characterization of the venom gland transcriptome for scorpion species belonging to the Vaejovidae family.

## Introduction

Scorpions are one of the oldest groups of living organisms on the earth with an estimated apparition over 400 million of years ago [1,2]. These arthropods gather around 1700 species, which are distributed in almost any place in the world [3,4]. Scorpions protect themselves against predators and capture preys by means of sophisticated venoms that contain a molecular arsenal of different toxins and other peptides, which are synthesized in and secreted from specialized glands. Scorpion venoms can be considered as true combinatorial libraries of peptides [5]. They contain toxins with lethal effects on humans [6] and potential therapeutic molecules, which have evolved under selective pressure [7]. As early as the 1960s venoms were already known as complex mixtures of dozens of other (i.e. not neurotoxic) peptide components. Furthermore, enzymatic and cytolytic activities of whole or fractionated venoms have been known at least since the 1920s [7]. Recently, scorpion venom peptides have been classified into two main groups: the disulfide-bridged peptides (DBPs), which affect membrane-bound ion channels[8–10] and the non-disulfide-bridged peptides (NDBPs), which show multiple activities [11–13]. DBPs contain three to four disulfide bridges which can be further sub-classified, into four different families depending on the type of membrane channels that they affect. These families of toxins in order of medical relevance are the ones that affect: Na^+^, K^+^, Ca^2+^ and Cl^−^ channels respectively [4]. These channels perform fundamental activities geared at regulating the normal cellular physiology in mammals. The perturbation of the regular activity of these channels caused by the binding of scorpion venom peptides can result in significant alterations leading to the well-known symptoms that appear during envenomation [14,15].

The fortuitous medical relevance of scorpion neurotoxins (the “killing component” with lethal effects on humans [16]), and their associated importance as pharmacological tools for the evaluation of ion channel’s function, has biased research priorities towards the characterization of these particular components of scorpion venoms [7]. However, the non-toxic (NDBPs) component of the venoms with potential therapeutic properties has just started to be characterized. Several NDBPs have been identified and functionally characterized only in the last decade. Some of the biological functions determined in NDBPs include: antimicrobial, antiviral, antimalarial, anticancer, hemolytic, anti-inflammatory, immune-modulatory and bradykinin potentiating activities [17]. Those activities, though clinically relevant, were frequently accompanied by low selectivity and high cytotoxicity in many of the reported NDBPs. This hindered their potential therapeutic applications [18,19]. For example, hadrurin, the first reported scorpion NDBP with antibacterial activity, inhibited the growth of Gram-positive and Gram-negative bacteria with a Minimal Inhibitory Concentration (MIC) of 10–50 μM, but it also resulted highly hemolytic to human erythrocytes at 30 μM [20]. A decade of research in the field of scorpion NDBPs has yielded several peptides with relatively mild cytotoxic effects and improved therapeutic indexes (the ratio between the toxic and the therapeutic doses), opening a window to their use as drug candidates [21].

Early methods for the identification of NDBPs were based on chromatographic separations combined with mass spectrometry. Further characterization included biological assays dedicated to determine the pharmacological properties of the potential therapeutic peptides [22]. These early biochemical characterizations were later complemented by Edman degradation peptide sequencing, although with technical limitations when sequencing lengthy peptides and/or with N-terminal modifications [20,23]. One of the major breakthroughs in scorpion venom peptidomics was the combination of molecular cloning with HPLC fractionation and mass spectrometry. This integration of technical tools even with the limitations imposed by limited N-terminal sequence information was circumvented by designing degenerate primers allowing the cloning of full-length cDNA sequences of the toxin precursor peptides [24].

The results obtained by several groups using cDNA libraries, revealed the existence of a rich biodiversity of components in the species studied. Apart from the peptides that modify ion-channel permeability, initially isolated and characterized by classical biochemical methods from species belonging to Buthidae family, many other components were identified, such as: factors that activate lipolysis, phospholipase A2, serine-proteases, metalloproteinases, protein homologs of tick salivary glands, precursors of cytolytic peptides, cysteine-rich peptides with no homology with common scorpion toxins and a great number of proteins and peptides deduced from the ESTs for which the function is still unknown [7,25–27]. Confirmation of these findings and new information about novel potential therapeutic peptides in Buthidae species has been recently reported by our group [28]. Furthermore, construction of cDNA libraries allowed the screening of several random cDNA clones, proving to be a successful strategy for the identification of several putative NDBPs reported elsewhere[13].

The determination of the transcriptomic profile of NDBPs provides additional information about the post-translational processing and the evolutionary characteristics of such peptides. These approaches are proving to be important tools for taxonomy as well [7]. Taken together, recent proteomic and transcriptomic analysis of scorpion venom and venom glands, have provided new data that have critically increased our knowledge about scorpion venom biology and on the cellular processes taking part on the highly specialized venom glands. Particular emphasis is made on novel components whose function, whether in the context of venom effects in susceptible animals or within the venom gland itself, has just started to be known [7].

Mass fingerprinting studies have revealed that NDBPs constitute more than 30% of all peptides present within scorpion venoms [7,29]. In spite of constituting a significant fraction of the total venom, this group of peptides has been poorly characterized when compared with DBPs that represent the majority of functionally characterized scorpion venom peptides reported in the literature. The importance of characterizing NDBPs is sustained by their biological and structural diversity associated to their multiple potential effects on cognate targets.

The Buthidae family, to which poisonous scorpions belong, makes up more than 50% of the approximately 1700 scorpion species currently known [30]. From approximately 800 Buthidae species only 34, are potentially dangerous to humans [6]. Non-Buthidae species have been largely neglected by toxicological research. Recently it has been demonstrated that even neglected lineages of scorpions are a rich source of novel biochemical components, which have evolved over millions of years to target specific ion channels in prey animals, but also new NDBPS with significant implications in therapeutics [31,32]. Non-Buthidae scorpions produce venoms with low or no toxic effects to mammals. The Vejovidae family is one of the non-Buthidae scorpion families that does not pose any risk for human health and which members can be localized in the North American subcontinent, mostly in Mexico [3]. The Vaejovidae family is composed of 17 genera, including *Smeringurus, Paruroctonus, Pseudouroctonus, Serradigitus*, and *Vaejovis*, among others. This last genus, *Vaejovis* (corr. Vejovis): 1836, C.L. Koch, is distributed from the southwestern United States to Guatemala, with the large majority of the 70 species inhabiting the Mexican territory [33–35]. In accordance with the classification proposed by Sissom in 2010, the *Vaejovis* genus is subdivided in 5 groups: *eusthenura, intrepidus, mexicanus, nitidulus* and *punctipalpi*, plus a group classified as *incertae sedis*. Examples of species belonging to some of the groups are *Vaejovis punctatus* (*eusthenura* group), *Vaejovis intrepidus* and *Vaejovis subcristatus* (*intrepidus* group), *Vaejovis mexicanus* (*mexicanus* group), among others.

In scorpions of the *Vaejovis* genus, the venom contains lesser amounts of ion channel toxins and higher amounts of NDBPs [36]. From two Mexican species belonging to the Vejovidae family (*V. mexicanus* and *V. subcristatus*), some NDBPs with therapeutic properties have been identified: Vejovine, with antibiotic activity against a broad spectrum of clinical isolates of bacteria from different genera [37]; Vm24, an immune-suppressive peptide selective to Kv1.3 potassium channels of human lymphocyte T cells [38,39]; VmCT1 and VmCT2, two antimicrobial peptides with a broad-spectrum activity against Gram negative and Gram positive bacteria [40]; and VsCT1 and VsCT2, two hemolytic peptides [40].

All this information as a whole is changing the paradigm of the study of scorpion venom toward a vision beyond their toxic properties. Every day new peptides with therapeutic properties are discovered. These results encourage to further study the venoms of non-Buthidae scorpions, in which NDBPs constitute a rich mine to be exploited. The detailed transcriptomic profile of the venom glands of species belonging to *Vaejovis* genus will retrieve valuable information that would help to identify new potential candidates to be used as therapeutic agents against different diseases. In this contribution, we report the generation and characterization of the cDNA libraries corresponding to four species of scorpions from the *Vaejovis* genus: *V. mexicanus*, *V. subcristatus*, *V. punctatus* and *V. intrepidus*.

## Materials and Methods

### 2.1. Biological Material

The scorpion specimens used in this work were collected in several locations in Mexico, under the official permit SGPA/DGVS/10638/11 by the Mexican Federal Government (issued by *Secretaría de Medio Ambiente y Recursos Naturales*, SEMARNAT). *V*. *mexicanus* and *V*. *punctatus* were collected in Cuernavaca, Morelos; *V*. *intrepidus* in Colima, Colima; and *V*. *punctatus* in Totalco, Veracruz.

The specimens were identified and classified in accordance with the available taxonomic keys [34,41]. The animals were kept in captivity, under standard conditions of temperature, humidity, and light and dark periods. They were periodically fed with crickets and supplied with water *ad libitum*.

### 2.2. cDNA Library Construction

Scorpions produce and store their venom only in the venom glands. These are a pair of well-delimited structures within the telson, which is the last segment of the metasome. For library construction, total RNA was extracted from telson macerates. For *V*. *punctatus* and *V*. *subcristatus* one specimen was used, whereas for *V*. *mexicanus* and *V*. *intrepidus* two telsons were dissected. Total RNA was extracted with the “SV Total RNA Isolation” Kit (Promega, Madison, WI) in accordance to the instructions from the manufacturer. Full-length cDNA libraries were prepared using the Creator SMART cDNA Library Construction Kit (CLONTECH Lab., Palo Alto, CA), designed to preferentially enrich for full-length cDNAs. For the first-strand cDNA synthesis, the provided oligonucleotides SMART IV (5’-AAGCAGTGGTATCAACGCAGAGTGGCCATTACGGCCGGG-3’) and CDS III/3’ PCR (5’-ATTCTAGAGGCCGAGGCGGCCGACATG-d(T)_30_N_-1_N-3’; with N = A, G, C, or T; N_-1_ = A, G or C) were used as primers. For cDNA amplification, the oligonucleotides 5’ PCR Primer (5’-AAGCAGTGGTATCAACGCAGAGT-3’) and CDS III/3’ PCR Primer were used. For both reactions, the conditions suggested by the manufacturer were followed. After the amplification, asymmetrical SfiI restriction sites (named SfiIA and SfiIB sites) flank the resulting cDNA molecules, allowing directional cloning. Following SfiI digestion, the cDNA was fractionated on CHROMA SPIN-400 columns to enrich the medium-sized fragments that correspond to the expected size of peptide-coding mRNAs. The selected fractions were pooled and concentrated by ethanol precipitation. The cDNA was ligated to the pDNR-LIB plasmid through the SfiIA and SfiIB sites, and the ligation was electroporated into electrocompetent *Escherichia coli* DH5α cells. Four full-length cDNA libraries were produced with this protocol. The titer of the non-amplified libraries was in the order of 1x10^6^ cfu/mL for *V*. *mexicanus*, *V*. *intrepidus* and *V*. *punctatus* and 1x10^5^ cfu/mL for *V*. *subcristatus*, all the libraries with 99% recombinant clones.

### 2.3. DNA Sequencing and Bioinformatic Analysis

Plasmids from selected colonies were isolated according to the standard alkaline lysis protocol [42]. The cloned inserts were sequenced on one direction with the T7 primer (5’-GTAATACGACTCACTATAGGG-3’) using an automatic machine (Model 3100, Applied Biosystems, Foster city, CA) according to the manufacturer’s instructions.

The nucleotide sequences obtained in this work were deposited in the GenBank (EST database: dbEST JZ818318-JZ818449). Phred scores were used to assess the sequence quality and to remove low-quality regions (end clipping) [43,44], with the window length set to 100 and the standard quality to 20. The cross_match utility was used to remove irrelevant vector and *E*. *coli* DNA sequences (http://www.phrap.com). ESTs that shared an identity of >95 out of 100 nucleotides were assembled in contiguous sequences with the CAP3 program (http://pbil.univ-lyon1.fr/cap3.php) [45]. All the bioinformatic analyses were performed online through the server of the Laboratorio de Biologia Molecular, Universidade de Brasilia (http://www.biomol.unb.br), using the default settings. The *Vaejovis mexicanus*, *V*. *intrepidus*, *V*. *punctatus* and *V*. *subcristatus* venom gland ESTs (clusters and singlets) were searched against the non-redundant nucleotide and protein databases using the blastn and blastx algorithms, respectively (http://www.ncbi.nlm.nih.gov/blast) with an e-value cutoff set to <10^–5^ to identify matches for the new ESTs. Additional search was performed with ORF Finder (Open Reading Frame Finder; http://www.ncbi.nlm.nih.gov/projects/gorf/), PROSITE (http://prosite.expasy.org/) and Pfam (http://pfam.sanger.ac.uk). Signal peptides for secreted proteins were predicted with the SignalP 4.0 program (http://www.cbs.dtu.dk/services/SignalP/). The possible propetide regions were predicted by using the Prop 1.0 server (http://www.cbs.dtu.dk/services/ProP/) and the SpiderP server (http://www.arachnoserver.org/spiderP). Multiple sequence alignments were performed with ClustalW2 (http://www.ebi.ac.uk/Tools/msa/clustalw2/), ClustalX 2.0 and T-Coffee (http://www.ebi.ac.uk/Tools/msa/tcoffee/). The consensus sequences were determined with CLC Sequence Viewer (v 6.8.1). The percentages of sequence identity were determined with LALIGN (http://embnet.vital-it.ch/software/LALIGN_form.html). The peptides chemical parameters, net charge and molecular weight were predicted with ProtParam (http://web.expasy.org/protparam/).

## Results and Discussion

### 3.1. Transcriptomic Analysis of the *Vaejovis* cDNA Libraries

For the four species used in this work, cDNA libraries were constructed and screened for sequences encoding venom components. Their titers ranged from hundreds of thousands (*V*. *subcristatus*) to millions (*V*. *mexicanus*, *V*. *intrepidus* y *V*. *punctatus*) of colony-forming units (cfu).

From the *V*. *punctatus* library 71 clones were sequenced, of which 28 ESTs were clustered in 11 contigs (corresponding to two or more ESTs) and 43 singlets (unique sequences). The ESTs were putatively classified in accordance with their similarity to reported sequences as 12 (16.9%) toxin genes, 12 (16.9%) antimicrobial peptides, 6 (8.5%) proteins involved in cellular processes, 6 (8.5%) proteins with unknown function, 1 (1.4%) La1-like peptide, 1 (1.4%) corresponded to other venom components, and 33 (46.5%) had no match with any sequence in the databases.

For *V*. *subcristatus*, 46 clones were sequenced. Twenty-four ESTs were grouped in 8 contigs and the remaning 22 were singlets. By sequence similarity, the ESTs were identified as possibly coding for 2 (4.3%) toxins, 6 (13%) antimicrobial peptides, 4 (8.7%) proteins involved in cellular processes, 12 (26.1%) proteins with undefined function, 1 (2.2%) La1-like peptide, 1 (2.2%) matched with other venom components, and 20 (43.5%) found no match in the databases.

Two hundred twenty-five clones were sequenced from the *V*. *mexicanus* library, resulting in 197 ESTs, clustered in 36 contigs and 61 singlets. Considering their possible function by homology, the ESTs were classified as 23 (11.7%) toxins, 119 (60.4%) antimicrobial peptides, 17 (8.6%) proteins involved in cellular processes, 17 (8.6%) proteins with undefined function, 1 (0.5%) La1-like peptide, 2 (1%) showed similarity with other venom components and 18 (9.1%) had no match with the sequences in the databases.

From the *V*. *intrepidus* library 103 clones were sequenced. Fifty-five ESTs were grouped into 17 contigs and the remaining 48 were singlets. Eleven (10.7%) matched with toxin sequences, 23 (22.3%) with antimicrobial peptides, 20 (19.4%) with proteins involved in cellular processes, 14 (13.6%) with proteins with undefined function, 4 (3.9%) with La1-like peptides, 2 (1.9%) with other venom components, and the remaining 29 (28.1%) found no homologs in the databases.

**Fig 1:**
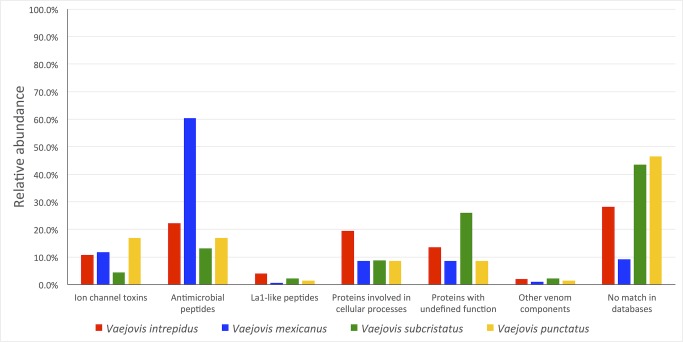
Relative distribution of the sequences found in the *V*. *intrepidus*, *V*. *mexicanus*, *V*. *subcristatus* and *V*. *punctatus* libraries with respect to the putative function of their encoded peptides.

Fig. 1 shows a comparison of the found relative abundance of the different peptide classes in the sequenced clones of the *Vaejovis* libraries. It is clear that, with respect to the remaining venom components, transcripts coding for ion channel-acting toxins are relatively scarce in the *Vaejovis* libraries. This is in accordance with previous findings [36].

### 3.2. Disulfide-bridged peptides found in the *Vaejovis* species

The venoms of the non-Buthidae scorpions contain non-disulfide bridged peptides as their main components [10,46–52], whereas some disulfide-bridged peptides can also be present. The sequence analysis performed with the cDNA libraries of the four *Vaejovis* species revealed the presence of precursor sequences coding for sodium, potassium and calcium channel toxins, plus some sequences corresponding to La1-like and scorpine-like peptides, all of which are rich in cysteines.

### 3.3. Sodium channels toxins (NaScTx)

**Fig 2:**
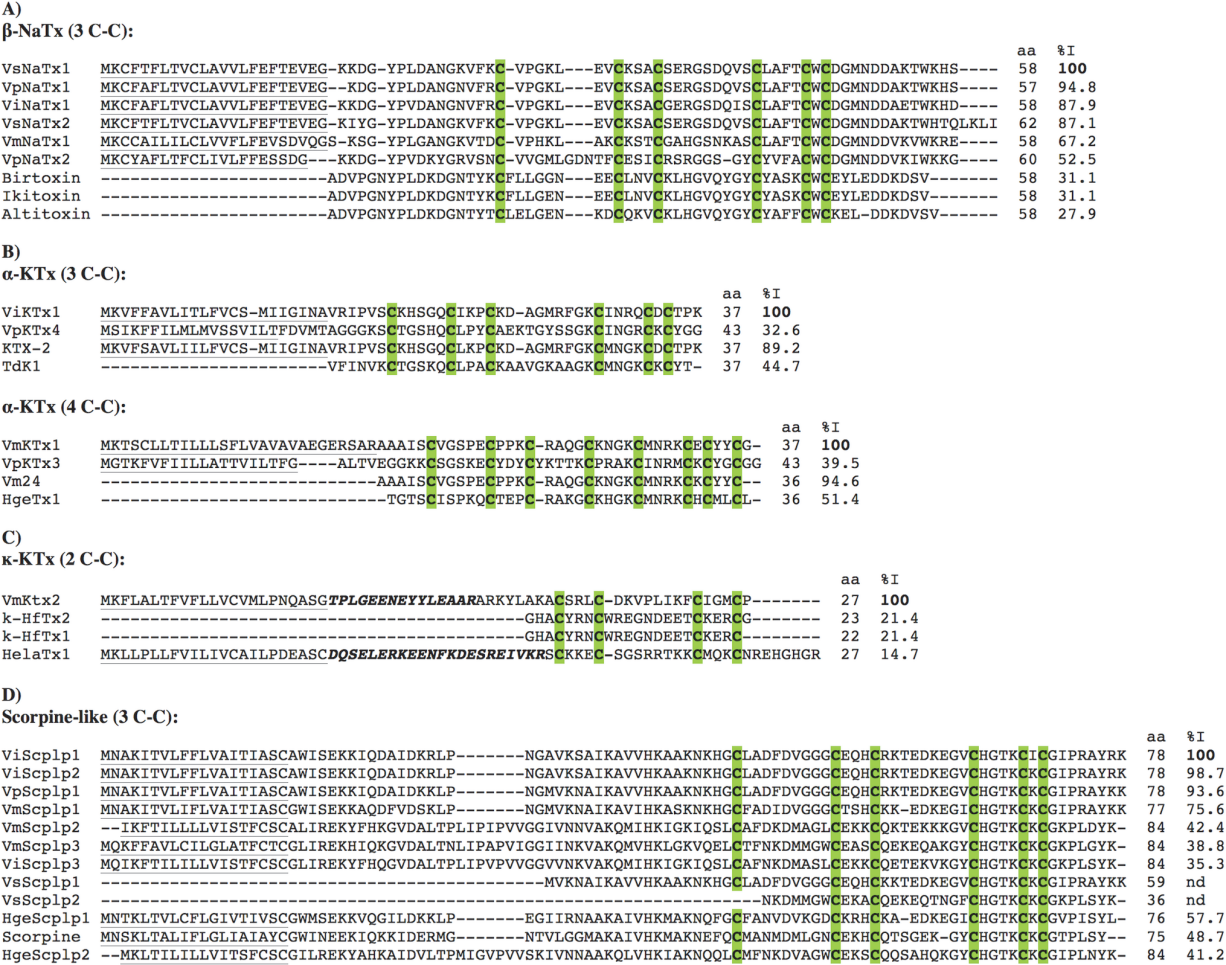
Putative toxin sequences derived from the precursors found in the *Vaejovis* libraries. The peptide length (aa) always refers to the confirmed (when the peptide has been isolated from the venom) or software-predicted mature peptides. The identity (%I) is always relative to the first sequence of the alignment, and considers only the mature peptide regions. When present, the signal peptides are shown underlined and the propeptides are in italics and bold. The conserved cysteine arrangement typical of each family is highlighted. A) Sequence alignment of the putative sodium channel toxins. The precursor sequences of ViNaTx1 from *V*. *intrepidus*, VpNaTx1 and VpNaTx2 from *V*. *punctatus*, VsNaTx1 and VsNaTx2 from *V*. *subcristatus*, and VmNaTx1 from *V*. *mexicanus* are compared to the known sodium channel-specific ß-toxins Birtoxin (UniProt:P58752), Ikitoxin (UniProt:P0C1B8) and Altitoxin (UniProt:P0C1B5) from *Parabuthus transvaalicus*. B) Comparative alignments of the sequences found that belong to the two families of potassium channel-specific α-toxins. First, the precursor sequences of the six-cysteines α-toxins ViKTx1 form *V*. *intrepidus* and VpKTx4 from *V*. *punctatus* aligned to KTX-2 (Kaliotoxin-2, UniProt:P45696) from *Androctonus australis* and TdK1 (UniProt:P59925) from *Tityus discrepans* as references. Second, the precursor sequences coding for the toxins belonging to the family of the eight-cysteines α-toxins VmKTx1 from *V*. *mexicanus* and VpKTx3 from *V*. *punctatus*, aligned to Vm24 (UniProt:P0DJ31) from *V*. *mexicanus* and HgeTx1 (UniProt:P84864) from *Hadrurus gertschi* as references. C) The precursor sequence of VmKTx2 from *V*. *mexicanus* compared to the precursor sequence of HelaTx1 (UniProt:P0DJ41) from *Heterometrus laoticus* and κ-HfTx1 (UniProt:P82850) plus κ-HfTx2 (UniProt:P82851) from *Heterometrus fulvipes*, all belonging to the family of potassium channel-specific κ-toxins. D) The scorpine-like sequences ViScplp1–3 from *V*. *intrepidus*, VmScplp1–3 from *V*. *mexicanus*, VpScplp1 from *V*. *punctatus*, and the partial sequences of VsScplp1–2 from *V*. *subcristatus* are aligned. For comparison, the sequences of scorpine (UniProt:P56972) from *P*. *imperator*, HgeScplp1 (UniProt:Q0GY40) and HgeScplp2 (UniProt:P0C8W5) from *H*. *gertschi* are also included in the alignment.

The scorpion toxins that alter the functionality of sodium ion channels are 58–76 amino acids-long peptides with a molecular weight of 6,500–8,500 Da [53]. These toxins are abundant in the venoms of the scorpions belonging to the Buthidae family and are responsible for the toxicity of several of those species on mammals, including humans [53]. In the non-Buthidae scorpions they are relatively less represented, and those found are generally active against insect sodium channels [46]. In the *V*. *intrepidus*, *V*. *punctatus*, *V*. *subcristatus*, and *V*. *mexicanus* libraries, sequences coding for putative NaScTx’s were found (ViNaTx1, VpNaTx1 and VpNaTx2, VsNaTx1 and VsNaTx2, and VmNaTx1, respectively). The mature peptides derived from the translated sequences contain six conserved cysteines and share sequence similarity with known scorpion sodium channel-acting ß-toxins (Fig. 2A). The mature *Vaejovis* NaScTx-like peptides display high sequence identity with each other, from 52.5% and up.

To date, the transcriptomic analyses performed with scorpions have revealed more than 140 sequences coding for putative NaScTx’s [16]. Some Buthidae scorpions apparently produce a large diversity of these toxins. For example, 39 NaScTx-like coding sequences were reported from a cDNA library of *Buthus occitanus israelis* [54] and 27 from a massive RNA-seq with *Centruroides noxius* [55]. In sharp contrast with this, the transcriptome analyses of non-Buthidae scorpions have yielded no putative NaScTx-coding sequences or just a few. The trascriptomes of *Hadrurus gertschi* [10], *Opisthacanthus cayaporum* [52], *Scorpiops jendeki* [49], *Heterometrus petersii* [50], *Pandinus cavimanus* [47] and *Scorpiops margerisonae* [31] revelaed no putative NaScTx-coding sequences, whereas those of *Scorpio maurus palmatus* [46] and *U*. *yaschenkoi* [48] returned only one each. Our analyses are in line with these reports, with one putative NaScTx found for *V*. *intrepidus* and *V*. *mexicanus*, and two found for *V*. *punctatus* and *V*. *subcristatus*. Since the NaScTx’s are known to be the main venom components responsible for the severe envenomation symptoms produced by the sting of the dangerous scorpion species [53], their low representativeness in the venoms of the Vaejovidae explain why none of the four species here studied are considered dangerous to humans [56].

### 3.4. Potassium channel toxins (KScTx)

Scorpion venoms are rich in toxins that affect potassium channels. They are classified in accordance to their primary sequence and their cysteine arrangement in four families: α-, β-, γ- and κ-KTx’s [57]. The α-KTx family is the most abundant in the scorpion venoms. These peptides are composed of 23–42 amino acids and adopt a typical cysteine-stabilized α/β fold with three or four disulfide bridges [58]. We found precursor sequences for putative α-KTx’s of the two kinds. First, those with three disulfide bridges: ViKTx1 from *V*. *intrepidus* and VpKTx4 from *V*. *punctatus*. An alignment of these possible toxins with other members of the α-KTx subfamily, is shown in Fig. 2B. Second, those with four disulfide bridges: VmKTx1 from *V*. *mexicanus* and VpKTx3 from *V*. *punctatus*. These are shown in Fig. 2B, aligned to two previously reported toxins of the same kind for comparison.

A precursor sequence coding for a putative κ-KTx was also found in the *V*. *mexicanus* library: VmKTx2. κ-KTx’s are toxins conformed by two parallel α-helixes connected by two disulfide bonds [16]. The precursor sequence for VmKTx2 contains a signal peptide, a propeptide and a predicted 27 amino acids-long mature peptide (Fig. 2C).

Our results are in correspondence with previous reports for non-Buthidae species. In the transcriptome of *H*. *gertschi* two α-KTx- and one β-KTx-coding precursors were found [10]. For *O*. *cayaporum* only one putative α-KTx was reported [52]. *S*. *jendeki* and *H*. *petersii* were more diverse, with eight [49] and four [50] transcripts respectively, all coding for putative α-KTx's. *P*. *cavimanus* produced three precursors, coding for one possible toxin of each of the α-, β-, and κ-KTx families [47]. *S*. *margerisonae* was shown to contain transcripts for two putative α-KTX's [31]. More recently, for *S*. *maurus palmatus* three transcripts were found, one for a putative α-KTx and two for possible κ-KTx’s [46].

Selective blockers of potassium ion channels have a remarkable therapeutic potential in the treatment of several pathological conditions, including autoimmunity. For example, the proliferation of auto-reactive T lymphocyte lineages can lead to autoimmune diseases, including multiple sclerosis or type I diabetes[59]. High affinity blockers of the Kv1.3 potassium channels can specifically inhibit the proliferation of effector memory T (T_EM_) cells [60]. Vm23 and Vm24, two α-KTx’s purified from the venom of *V*. *mexicanus* are potent blockers of the human Kv1.3 channel [38]. The fact that we were able to identify cDNA sequences coding for yet undescribed putative KTx’s from the *Vaejovis* libraries reflects the value that this genus has a source of bioactive compounds with medical importance.

### 3.5. Scorpine-like peptides (Scplp)

Scorpine is an interesting peptide isolated from the *Pandinus imperator* venom. Its carboxyl-terminal region shows sequence similarity with the β-KTx family, with the typical cysteine-stabilized α/β fold, with 3 disulfide bridges. Its amino-terminal region, on the other side, is similar to peptides of the cecropin family. Scorpine was shown to display antibacterial activity and potent inhibitory effects on the development of *Plasmodium berghei* at the ookinete and gamete stages [61]. Scorpine homologs were thereafter discovered for *Opistophthalmus carinatus* [62], *Heterometrus laoticus* [63] and *H*. *gertschi* [10]. From the last species, HgScplp1 showed antibacterial activity against *Bacillus subtilis* and cytolitic activity on *Xenopus laevis* oocytes. When the N-terminal region of HgScplp1 was cleaved with cyanogen bromide, the remaining peptide displayed itself as an effective blocker of the Kv1.1, Kv1.2 and Kv1.3 channels [64]. This dual activity is unique to scorpine-like peptides, which can therefore be considered as modular peptides.

In the *Vaejovis* transcriptomes several scorpine-like sequences were found: ViScplp1, ViScplp2 and ViScplp3 in *V*. *intrepidus*; VmScplp1, VmScplp2 and VmScplp3 in *V*. *mexicanus*; VpScplp1 in *V*. *punctatus*; and the partial sequences of VsScplp1 and VsScplp2 in *V*. *subcristatus*. The last two sequences were not considered for sequence analysis, since they are incomplete, but the remaining precursor sequences share some degree of sequence identity, mainly driven by the C-terminal CS-α/β motif (see the %I in Fig. 2D). An interesting pattern emerges when only the N-terminal region is considered. In this case, some of the here reported sequences group with Scorpine and HgScplp1 (ViScplp1, ViScplp2, VmScplp1 and VpScplp1) to conform a short-chain clade, whereas others (ViScplp3, VmScplp2 and VmScplp3) share N-terminal similarity with HgScplp2 and conform a long-chain clade. It is remarkable that (as in the case of *H*. *gertschi*), both short- and long-chain scorpine-like peptides can be found in the same *Vaejovis* species. Whether this has any biological implications remains to be determined.

### 3.6. Calcins

The scorpion toxins that affect calcium channels are referred to as calcins. They are structurally characterized by the presence of an inhibitor cystine knot (ICK) motif, a folding that puts them structurally apart from the sodium, potassium and chloride channel toxins [65]. Calcins can penetrate the cell membranes and affect the ryanodine receptors (RyR) of the endoplasmic and sarcoplasmic reticula of the cardiac and skeletal muscle [66,67]. The alterations in the calcium potentials result in muscular paralysis, which contributes to the immobilization of preys [66].

The first RyR-specific activity recorded for scorpion venom components was discovered in a gel-filtration fraction obtained from the venom of *Buthotus hottentota* [68]. After that, calcins with high affinity for the RyR were purified from the *P*. *imperator* venom: imperatoxins IpTxa and IpTxi [67]. Other peptides were also characterized from different venoms, such as ryanotoxin from *Buthotus judaicus* [69] and the toxins Bmk-AS and Bmk-AS-1 from *Buthus martensii* Karsch [70].

**Fig 3:**
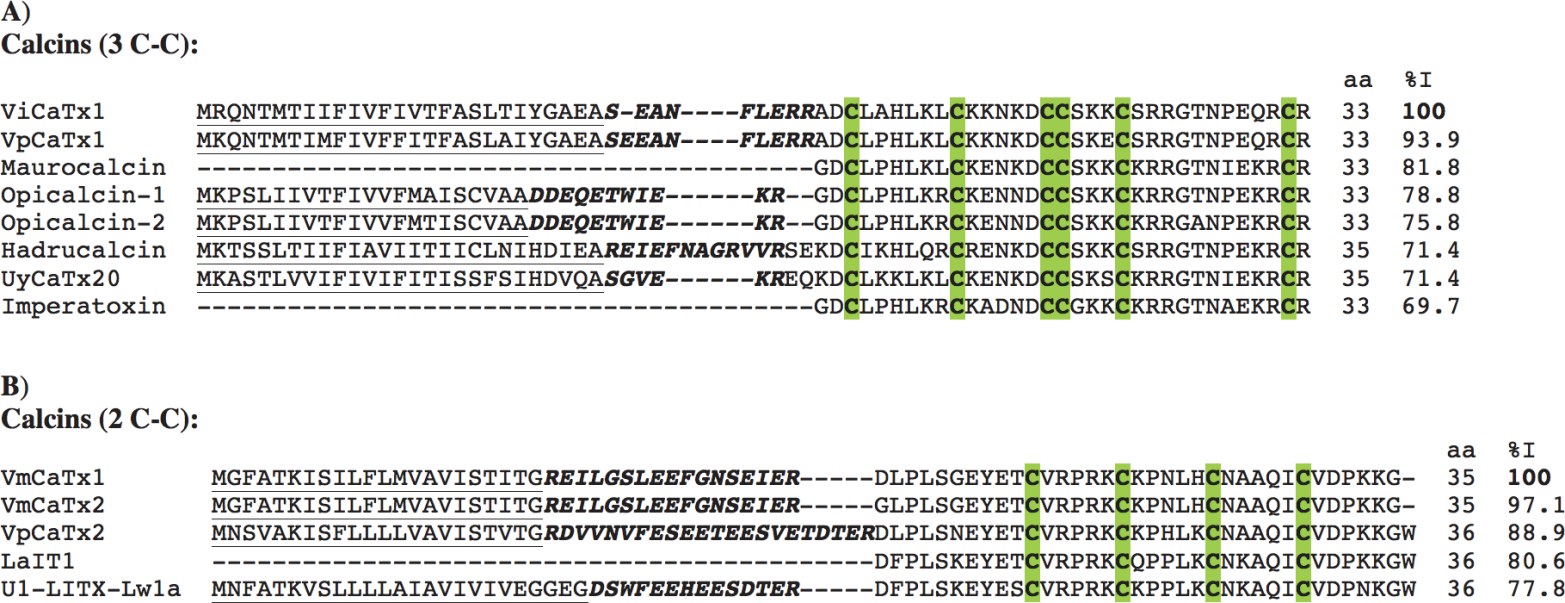
Putative calcium channel toxins found in *Vaejovis*. The peptide length (aa) always refers to the confirmed (when the peptide has been isolated from the venom) or software-predicted mature peptides. The given percentages of identity (%I) correspond to the mature peptides only, and refer to the first sequence. The predicted signal peptides are shown underlined, the propeptides in italics and bold. **A**) Sequence alignment of the three-disulfide-bridged putative calcinsVpCaTx1 from *V*. *punctatus* and ViCaTx1 from *V*. *intrepidus* with imperatoxin (UniProt:P59868) from *P*. *imperator*, maurocalcin (UniProt:P60254) from *S*. *maurus palmatus*, opicalcin-1 (UniProt:P60252) and opicalcin-2 (UniProt:P60253) from *Opistophthalmus carinatus*, UyCaTx20 (Calcium-channel toxin-like 20, UniProt:AGA82762) from *U*. *yaschenkoi* and hadrucalcin (UniProt:B8QG00) from *H*. *gertschi*. **B**) The precursors of the two-disulfide-bridged putative calcins VmCaTx1 and VmCaTx2 from *V*. *mexicanus* and VpCaTx2 from *V*. *punctatus*, are compared to previously reported sequences of their kind: LaIT1 (UniProt:P0C5F2) from *L*. *australasiae*, and U_1_-LITX-Lw1a (UniProt:P0DJ08) from *L*. *waigiensis*.

In the *Vaejovis* libraries we found two precursor sequences coding for putative calcins with three disulfide bridges: VpCaTx1 from *V*. *punctatus* and ViCaTx1 from *V*. *intrepidus*. Both toxins are composed of 33 amino acids, with 6 cysteines. They show high similarity with previously reported calcins, such as imperatoxin A from *P*. *imperator* [67], maurocalcin from *S*. *maurus palmatus* [71], hadrucalcin from *H*. *gertschi* [66], among others (Fig. 3A). The two *Vaejovis* calcins differ only by two residues, and share 70% identity with imperatoxin A, the most studied calcin to date.

Three precursors coding for possible calcins with two disulfide bridges were also found: VmCaTx1 and VmCaTx2 from *V*. *mexicanus*, and VpCaTx2 from *V*. *punctatus*. They display high sequence identity with the insect toxin LaIT1 from *Liocheles australasiae* [72] and toxin U_1_-LITX-Lw1a (ϕ-liotoxin-Lw1a) from *Liocheles waigiensis* [73] (Fig. 3B). The *Liocheles* toxins bind to and modulate the activity of RyR1 and RyR2 ryanodine receptors. They are reported to adopt the structural fold known as disulfide-directed hairpin (DDH) motif, a proposed evolutionary precursor of the three-disulfide ICK [73].

The putative calcins here reported are yet to be purified from the parental scorpions venoms. If they are indeed active on the RyR, they could become valuable tools for the study of these calcium channels.

### 3.7. La1-like peptides (La1lp)

La1-like peptides are cysteine-rich peptides that have thus far been found most frequently in the venoms of scorpions that are not toxic to mammals. La1, the first described peptide of this family, was reported as the most abundant component of the venom of the scorpion *L*. *australasiae* [74]. The La1 peptide was tested with crickets but showed no toxicity. Lately, La1-like peptides have been found in other scorpion venoms, as *O*. *cayaporum* [52], *U*. *yashenkoi* [48], *P*. *cavimanus* [47], *S*. *margerisonae* [31], *H*. *petersii* [50] and *S*. *maurus palmatus* [46]. Six different transcripts coding for putative La1-like peptides were found for *S*. *jendeki* [49]. Interestingly, similar peptides have also been found in scorpions belonging to the Buthidae family: *Lychas mucronatus* and *Isometrus maculatus* [31].

The La1-like peptides are presumed to possess a single Von Willebrand factor type C domain (VWC), a structural feature shared by several eukaryotic proteins with cell biology functions [75,76], although this has not been proven [77].

**Fig 4:**
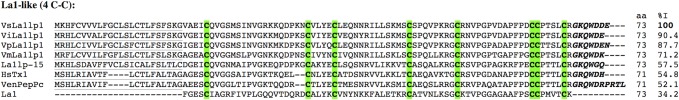
Sequence alignment of the La1-like peptides found in the Vaejovis libraries. The precursor sequences of VmLa1lp1 from *V*. *mexicanus*, VsLa1lp1 from *V*. *subcristatus* and VpLa1lp1 from *V*. *punctatus* are aligned to the previously reported precursors of La1lp-15 (UniProt:AGA82761) from *U*. *yaschenkoi*, HsTx1 (UniProt:K7WMX6) from *H*. *spinifer*, VenPepPc (UniProt:H2CYP1) from *P*. *cavimanus*, and the mature La1 (UniProt:P0C5F3) from *L*. *australasiae*. The peptide length (aa) always refers to the confirmed (when the peptide has been isolated from the venom) or software-predicted mature peptides. The given percentages of identity (%I) correspond to the mature peptides only, and are relative to the first sequence. The predicted signal peptides are shown underlined, the propeptides in italics and bold.

Four precursors of La1-like peptides were found in the *Vaejovis* libraries: VmLa1lp1 from *V*. *mexicanus*, VsLa1lp1 from *V*. *subcristatus* and VpLa1lp1 from *V*. *punctatus* and ViLa1lp1 from *V*. *intrepidus*. They share over 70% sequence identity, and present high similarity with the other previously described La1-like peptides, as shown in Fig. 4. They all present the VWC domain with conserved 8 cysteines. The purified mature La1 was confirmed to be amidated [74]. In the sequences encoded by all the precursors, right after the positions equivalent to the last amino acid of La1, a potential amidation substrate (GK or GR) is present. We therefore assume that all La1-like peptides are amidated and that the precursors contain a propeptide (post-peptide) after the mature region, as indicated in Fig. 4.

The activity of the La1-like peptides remains to be determined. This is a relevant subject due to their seemingly wide distribution in the Scorpiones order.

### 3.8. Non-disulfide-bridged peptides (NDBPs)

The interest for the scorpion venom non-disulfide-bridged peptides (NDBPs) has increased in the last decade. This is due to their structural diversity and the multiple functions that they can perform, which highlights them as potential candidates for many applications, including drug development. They are 13–56 amino acids-long cationic peptides with random coil conformation in water, but in cell membrane-mimicking environments, they adopt an amphipathic α-helical structure. More than 40 scorpion NDBPs have been functionally characterized to date [17]. Among them, more than 30 display antimicrobial and cytolytic properties. They can inhibit the growth of Gram-negative and Gram-positive strains of bacteria, and are therefore considered to be the organism’s first line of defense against pathogens. Some of these peptides have been isolated from scorpion venoms by biochemical techniques, but an increasing number of NDBP sequences have been derived from the precursor sequences found in cDNA libraries [78].

Since the first report of a scorpion antimicrobial peptide, hadrurin from *Hadrurus aztecus* (now known as *H*. *gertschi)* [20], we have found several other scorpion NDBPs, from both Buthidae and non-Buthidae species [37,40,48,79]. We have already reported two NDBPs from the *Vaejovis* genus, vejovine a peptide with 47 amino acids isolated from the venom of *V*. *mexicanus* and VmCT1 a short peptide derived from a cDNA library of *V*. *mexicanus* [37,40]. The analysis of the cDNA libraries constructed for the four *Vaejovis* species revealed the presence of several new peptides. They belong to the subfamilies NDBP-2 and NDBP-4, according to the classification recently proposed by Almaaytah and Albas [17].

### 3.9. Short antimicrobial peptides (NDBP-4)

The members of the NDBP-4 subfamily are short antimicrobial peptides. They are derived from their much longer peptidic precursors by posttranslational processing of a signal peptide and a carboxy-terminal propeptide region (post-peptide) that flank the mature peptide. The signal peptide has 23–24 amino acids and is the most conserved region of the precursor sequence. The conserved GKR sequence delimits the mature region. It is a putative protease cleavage site, probably for a peptidylglycine alpha-amidating monooxygenase, the kind of enzyme that renders the mature peptide amidated in the carboxyl-terminus [80]. The propeptide region has 32–42 amino acids and is rich in Glu and Asp residues. The mature peptide contains only 13 amino acids, most of them hydrophobic. Though there is some sequence conservation among the mature members of the NDBP-4 family, the region of the mature sequence is the least conserved throughout the precursor sequences. The secondary structure prediction analysis shows that these peptides can fold into an amphipathic α-helix. Several mature peptides from this family have been tested for their functionality and shown to display antimicrobial and hemolytic activities. For example, IsCT (CT stands for cytotoxic), a peptide isolated from the venom of *Opisthacanthus madagascariensis*, has been shown to display antibacterial activity against Gram-negative and Gram-positive strains of bacteria with minimum inhibitory concentrations (MIC) in the range of 0.7–17.0 μM [81]. To date, about 24 different peptides with sequence similarities to IsCTs have been tested for activity. They have been found in all scorpion families thus far studied [17]. We have previously reported the characterization of two homologs of IsCT derived from a cDNA library of *V*. *mexicanus*: VmCT1 and VmCT2 [40]. Both peptides were able to inhibit the growth of Gram-negative and Gram-positive strains of bacteria with MICs of 5–25 μM. VmCT2 was mildly hemolytic at its MIC range, but showed strong hemolysis at higher concentrations. VmCT1 produced only mild (12%) hemolysis even at 50 μM, far beyond the MIC range.

**Fig 5:**
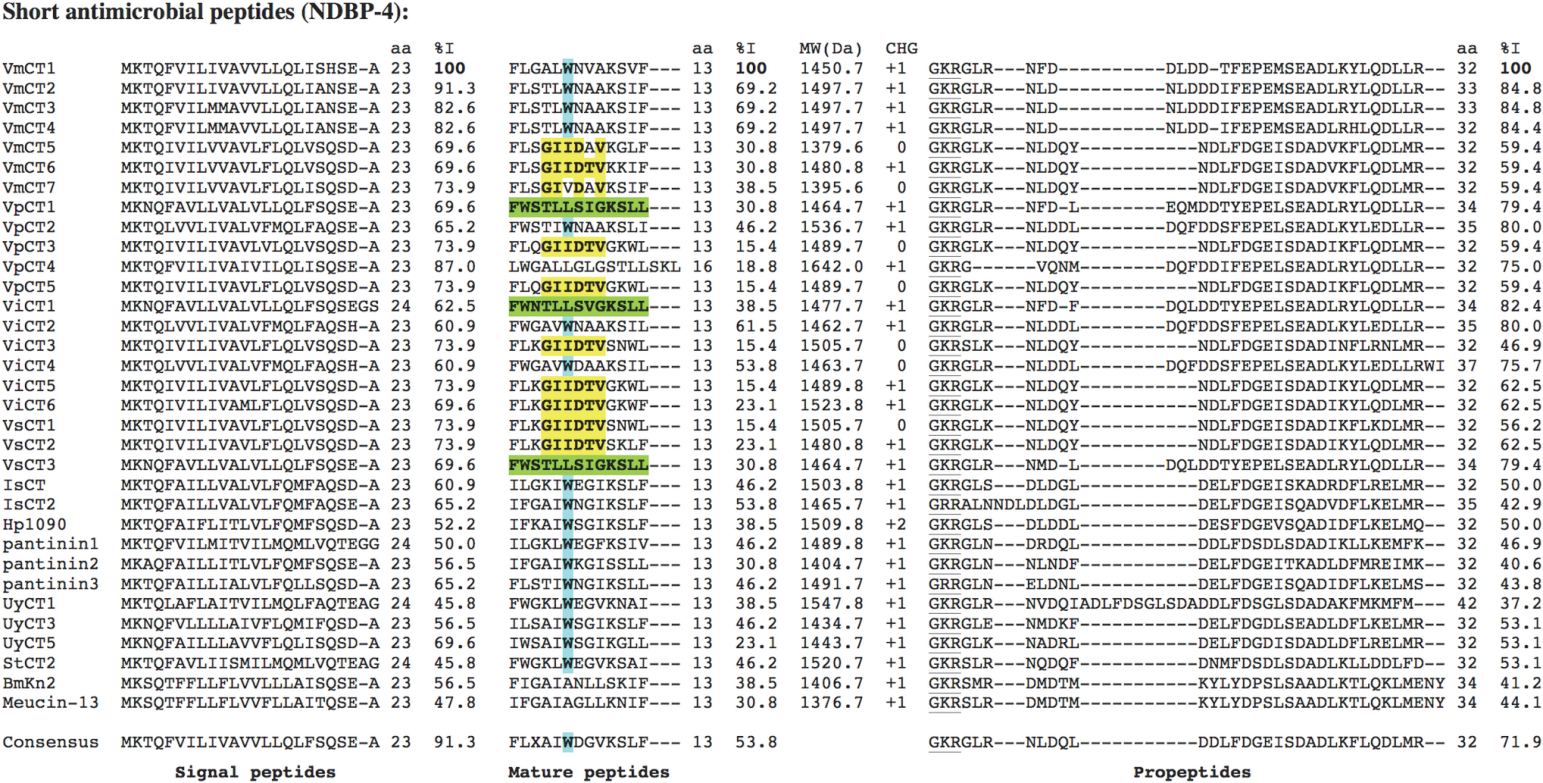
Multiple alignment of the sequences corresponding to the peptidic precursors belonging to the NDBP-4 subfamily found in the *Vaejovis* libraries. Separate alignments are shown for the signal peptides, the mature peptides and the propeptides, with the number of residues (aa) and the percentage of identity (%I) indicated for each segment. For the mature peptide the prominent sequence features are highlighted with colors and in bold typeface (see text) and the theoretical molecular weight (MW) and net charge (CHG) are also given. In the propeptide region, the post-translational cleavage and amidation substrate sequence is underlined. For comparison, the sequences of IsCT (UniProt:Q8MMJ7.1), IsCT2 (UniProt:Q8MTX2.1), UyCT1 (UniProt:L0GCV8.1), UyCT3 (UniProt:L0GCI6.1), UyCT5 (UniProt:L0GAZ8.1), Hp1090 (UniProt:P0DJ02.1), Pantinin 1 (UniProt:R4JNJ5), Pantinin 2 (UniProt:R4JQZ0), Pantinin 3 (UniProt:R4JJN6), Meucin-13 (UniProt:E4VP07.1), BmKn2(UniProt:Q6JQN2.1) and StCT2 (UniProt:P0DJO4.1) were used.

The analysis of the cDNA libraries made with four *Vaejovis* species revealed the presence of several precursors for peptides belonging to the NDBP-4 subfamily. From the *V*. *mexicanus* library, 12 clusters were found (9 contigs and 3 singlets); for *V*. *intrepidus*, 7 clusters (3 contigs and 4 singlets); for *V*. *punctatus*, 5 clusters (2 contigs and 3 singlets); and for *V*. *subcristatus*, 3 clusters (3 contigs). Fig. 5 shows the alignment of the sequences found in the four *Vaejovis* libraries with the precursors of some members of the NDBP-4 subfamily, which mature peptides have been functionally characterized. The alignment shows that the region of highest identity corresponds to the signal peptide, while the region of the mature peptide is the one with the lowest identity. All the *Vaejovis* propeptides show a high content of acidic amino acids, and present the conserved GKR sequence at their amino-terminus. Some sequence patterns are noticeable in the *Vaejovis* mature peptides that allow distinguishing three types. First, some sequences present the GIIDTV motif, which is found in at least one sequence per species. Second, some others present a tryptophan residue at position 6, which has been proposed to play an important role in the structural properties of these peptides as well as in their antibacterial activities [40]. Finally, the sequences VpCT1, ViCT1 and VsCT3, present a pair of leucines at positions 5 and 6, plus another pair at positions 12 and 13. The sequence of VpCT4 is remarkable since it has a mature peptide of 16 amino acids, being the only peptide of this subfamily with more than 13 amino acids found in the *Vaejovis* libraries. Note that in Fig. 5 the sequences here reported are not ordered in accordance to the percentage of identity determined for any particular region of the precursors. They are instead sequentially ordered by species, so the diversity of the above mentioned sequence landmarks within each species is more evident.

### 3.10. Long-chain multifunctional peptides (NDBP-2)

**Fig 6:**
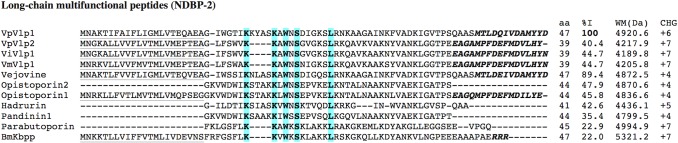
Multiple alignment of the sequences corresponding to the precursors of the peptides belonging to the NDBP-2 subfamily found in the *Vaejovis* libraries. The peptide length (aa) refers to the confirmed (when the peptide has been isolated from the venom) or software-predicted mature peptides. The identity (%I) is relative to the first sequence of the alignment, and considers only the mature peptide region. When present, the signal peptides are shown underlined and the propeptides are in italics and bold typeface. The theoretical molecular weight (MW) and net charge (CHG) are also given for each mature peptide. Fully conserved residues are highlighted with color and bold. For comparison purposes, the sequences of vejovine (UniProt:ADZ24463.1), hadrurin (UniProt:P82656.1), opistoporin-1 (UniProt:P83313.2), opistoporin-2 (UniProt:P83314.1), pandinin1 (UniProt:P83239.1), parabutoporin (UniProt:P83312.1), and BmKbpp (UniProt:Q9Y0X4.1) were used.

The NDBP-2 subfamily groups long-chain multifunctional peptides. Members of this subfamily have been shown to display antimicrobial, bradykinin-potentiating, insecticidal and anticancer activities. To the NDBP-2 subfamily belong peptides like hadrurin, parabutoporin, pandinin-1 and the opistoporins [17]. They are also produced as larger precursors that suffer posttranslational processing. The structure of these precursors is similar to that of the above-described NDBP-4 subfamily. Sequentially, the amino-terminal signal peptide is followed by the mature peptide and then, a propeptide. Signal peptides typically contain 22 amino acids, mature peptides 39–50, and propeptides 13–17. Mature peptides are rich in Lys and Arg residues, so they are usually highly charged cationic peptides. It was recently reported that vejovine, from *V*. *mexicanus*, is able to inhibit the growth of multiresistant Gram-negative bacterial strains from clinical isolates (*Pseudomonas aeruginosa*, *Klebsiella pneumoniae*, *Escherichia coli*, *Enterobacter cloacae* y *Acinetobacter baumanii*) with MICs of 4.4–50.0 μM. Here we report four new peptides belonging to the NDBP-2 subfamily that were derived from sequences found in the *Vaejovis* cDNA libraries. Fig. 6 shows the alignment of the precursors of these peptides with vejovine and other members of the NDBP-2 subfamily. Two distinct sequence types can be distinguished in the *Vaejovis* peptides. On one side, vejovine and VpVlp1 (Vlp stands for vejovine-like peptide) are larger peptides that show a high percentage of identity, with only 5 differences in the 47-amino acids sequence. On the other, VpVlp2, ViVlp1 and VmVlp1 are shorter, 39-amino acids peptides, that are less related to vejovine, but display a high percentage of identity (89.7–88.5%, not shown in Fig. 6) with each other. It is remarkable that these last peptides are the shortest members of the NDBP-2 family, while at the same time they have a large positive net charge of +7, found only in larger peptides, like parabutoporin and BmKbpp. They thus represent the NDBPs with the largest known density of positive charges. Peptides with increased positive charge have been shown to be more effective as antimicrobials (but also more hemolytic) [82].

## Concluding Remarks

This communication reports the first approach to the transcriptome analysis of scorpion species belonging to the Vaejovidae family. Many new unknown sequences were identified, but in general, these results confirm the previously observed predominance of non-disulfide bound peptides in species of scorpions distinct from those classical cysteine-rich channel-acting toxins found in the venoms of Buthidae species. In addition, it also shows that the non-Buthidae species have a rich diversity of components present in their venoms. Some of the new sequences are currently being assayed to assess their biological activity. Some of these peptides belong to families of previously characterized scorpion venom components that have shown potential application in fields of medical relevance, e.g. the development of new antibiotics and antimalarial drugs.
